# A size-shrinkable matrix metallopeptidase-2-sensitive delivery nanosystem improves the penetration of human programmed death-ligand 1 siRNA into lung-tumor spheroids

**DOI:** 10.1080/10717544.2021.1931560

**Published:** 2021-06-02

**Authors:** Jiaolin Wen, Neng Qiu, Zejiang Zhu, Peng Bai, Mengshi Hu, Wenyan Qi, Yan Liu, Ailin Wei, Lijuan Chen

**Affiliations:** a Laboratory of Natural Product Drugs, State Key Laboratory of Biotherapy and Cancer Center, West China Hospital, Sichuan University, Chengdu, China; b Department of Chemical & Pharmaceutical Engineering, College of Materials and Chemistry and Chemical Engineering, Chengdu University of Technology, Chengdu, China; c Guang’an People’s Hospital, Guang’an, China

**Keywords:** Programmed death-ligand 1, small-interfering RNA, hyaluronic acid, polyethyleneimine, matrix metalloproteinase-2

## Abstract

Given the maturation of small-interfering RNA (siRNA) techniques with nanotechnology, and because overexpression of human programmed death-ligand 1 (PD-L1) is crucial for T cell inactivation and immunosuppression of the tumor microenvironment, application of siRNA–PD-L1 has demonstrated positive progress in preclinical studies; however, the limited penetration of this compound into solid tumors remains a challenge. To decrease PD-L1 expression and increase the penetration efficacy of solid tumors, we synthesized a novel tumor-microenvironment-sensitive delivery polymer by conjugating hyaluronic acid (HA) to polyethyleneimine (PEI), with a matrix metalloproteinase-2 (MMP-2)-sensitive peptide acting as the linker (HA-P-PEI), for use in delivery of PD-L1–siRNA. Concurrent synthesis of a linker-less HA-PEI compound allowed confirmation that negatively charged siRNA can be complexed onto the positively charged HA-PEI and HA-P-PEI compounds to form nanoparticles with the same particle size and uniform distribution with serum stability. We found that the size of the HA-P-PEI/siRNA nanoparticles decreased to <10 nm upon addition of MMP-2, and that H1975 cells overexpressing CD44, PD-L1, and MMP-2 aided confirmation of the delivery efficacy of the HA-P-PEI/siRNA nanocomplexes. Additionally, the use of HA-P-PEI caused less cytotoxicity than PEI alone, demonstrating its high cellular uptake. Moreover, pretreatment with MMP-2 increased nanocomplex tumor permeability, and western blot showed that HA-P-PEI/PD-L1–siRNA efficiently downregulated the PD-L1 expression in H1975 cells. These results demonstrated a novel approach for siRNA delivery and tumor penetration for future clinical applications in cancer treatment.

## Introduction

Human programmed death-ligand 1 (PD-L1; B7H1) overexpression in tumor cells reduces recognition by T cells and promotes both tumorigenesis and invasion (Teo et al., 2015; Zou et al., 2016; Schalper et al., 2017). PD-L1 plays an immunosuppressive role by binding to PD-1 in T cells, with blockage of this interaction capable of effectively reversing T cell inhibition and avoiding immunosuppression in the tumor microenvironment (Benson et al., 2010; Yu et al., 2019). The United States Food and Drug Administration has approved several PD-L1 antibody drugs for Parkinson’s disease, including altizolumab, avilumab, and duvarumab (Chen et al., 2016), for clinical use. For patients with lung cancer, immunotherapy involving blockage of the PD-1–PD-L1 interaction has been demonstrated as first-line therapy (Suresh et al., 2020). Recently, progress in nanotechnology had increased interest in potential applications with small-interfering (si)RNA technology. In 2004, phase I clinical trials of an siRNA-based treatment for a type of eye disease were conducted (Whelan, 2005; Castanotto and Rossi, 2009), and the first targeted nanoparticle-delivery system for solid tumor patients entered clinical trials, marking the beginning of systematic application of siRNAs in solid tumours (Davis et al., 2010). Additionally, applications of siRNA–PD-L1 demonstrated positive progress in preclinical studies, where PD-L1–siRNA lipid-nanoparticle therapy increased the proliferation of natural killer cells and antigen-specific CD8+ T cells to enhance their killing and memory functions (Gato-Cañas et al., 2017). Moreover, previous studies showed that PD-L1 is involved in intracellular anti-apoptotic signals and affects the proliferation, apoptosis, and migration of tumor cells (Clark et al., 2016) and can transmit anti-apoptotic signals to tumor cells, thereby helping them avoid interferon-induced cell death (Li et al., 2017; Dong et al., 2018). Therefore, reexamining the downregulation of PD-L1 expression is crucial.

Polyethyleneimine (PEI) is widely used as a hydrophilic and positively charged polymer material for gene therapy. The main chain of the polymer can interact with the anionic phosphate of the siRNA through electrostatic interactions to form nanometer complexes with sizes ranging from 100 nm to 1000 nm (Günther et al., 2011). Moreover, degradation of siRNA by enzymes can be prevented after complexation to promote cellular uptake through endocytosis (Varkouhi et al., 2011). The disadvantage of PEI is its cytotoxicity and non-biodegradability, despite its high level of transfection efficiency (Liu et al., 2014). However, functionalization with polyethylene glycol (PEG) and hyaluronic acid (HA) can reduce PEI toxicity. HA is a nontoxic, non-immunogenic, negatively charged natural compound produced by the human body and that is also rich in carboxyl groups. The combination of polyethylene imide and HA promotes electrostatic neutralization of the nanoparticles. Additionally, HA contributes to the formation of protective hydrophilic surfaces, indicating that PEG and HA coupling can facilitate the passive targeting efficacy of nanomedicines through an enhanced permeability and retention effect, whereas nanoparticle permeability can be hindered by relatively large particle size (Cabral et al., 2011).

Strategies based on the tumor-microenvironmental response are considered ‘intelligent’ and have achieved favorable results in drug delivery (Zhang et al., 2013). The overexpression of matrix metalloproteinases (MMPs) is more significant than other anomalous features in tumors and can be used as a ‘smart’ drug-delivery and tumor-targeting compound. Furthermore, MMP systems can accurately regulate the release of drugs at different levels. Among MMP substrates, synthetic variants mainly comprise short linear peptides that are superior to natural micromolecular proteins and can directly couple with nanoparticles (Turk et al., 2001; Tu and Zhu, 2016). Members of the MMP family, including MMP-2 and MMP-9, are overexpressed in many cancer types (Egeblad and Werb, 2002) and can promote the destruction of extracellular matrix and play a critical role in tumor invasion and metastasis. Therefore, the development of MMP-2-sensitive tumor-imaging probes and delivery systems has previously been undertaken for cancer-specific therapeutics (Bremer et al., 2001; Ruan et al., 2015; Han et al., 2017).

Here, we synthesized a tumor microenvironment-sensitive delivery polymer by conjugating HA to cationic PEI along with an MMP-2-sensitive peptide (P; GPLGLAGC) (Han et al., 2017) linker (HA-P-PEI) to deliver the PD-L1–siRNA into H1975 cells. Additionally, we synthesized a linker-less HA-PEI variant to allow evaluation of both nanocarriers prepared as spheroids with the same particle size and a uniform distribution following complexation with PD-L1–siRNA.

## Experimental

### Materials

Sodium HA (20 kDa) was obtained from Lifecore Biomedical Inc. (Chaska, MN, USA), N-(2-aminoethyl) maleimide hydrochloride (AEM), and branched PEI (25 kDa) was obtained from Sigma-Aldrich (St. Louis, MO, USA). The oligopeptide GPLGLAGC(PLG) was synthesized by Shanghai Sangon Biotech Co., Ltd. (Shanghai, China), and negative control (NC) siRNA, Cy3-siRNA (Cy3-conjugated NC siRNA at the 5′-end), fluorescein amidite (FAM)-siRNA (FAM-conjugated NC siRNA at the 5′-end), and PD-L1–siRNA (Sense: UUCUCCGAACGUGUCACGUTT; and antisense: ACGUGACACGUUCGGAGAATT) (Liu, Cao, et al., 2019) were synthesized by Shanghai GenePharma Co., Ltd. (Shanghai, China). 1-Ethyl-3-(3-dimethylaminopropyl) carbodiimide (EDC) and 1-hydroxybenzotriazole (HOBt) were obtained from Sichuan Shuyan Biotechnology Co., Ltd. (Sichuan, China), and trypsin were purchased from Hyclone (Provo, UT, USA). MMP-2 was purchased from Sigma-Aldrich, and phycoerythrin (PE)-conjugated anti-CD274 (PD-L1; B7-H1) and allophycocyanin (APC)-conjugated anti-CD44 were purchased from eBioscience (San Diego, CA, USA).

### HA-PEI and HA-P-PEI synthesis

For HA-PEI synthesis (Jiang et al., 2008), 20 mg HA and 402 mg PEI were separately dissolved in pure water (10 mL), followed by mixing of the solutions at pH 6.5. EDC (40 mg) and HOBT (28 mg) were dissolved in a pure water/DMSO (500 µL/500 µL) solution, which was slowly added to the prepared HA/PEI solution. The reaction mixtures were stirred at room temperature for 24 h with the pH adjusted to 7.0. HA-PEI conjugates were then purified by dialysis (30 kDa) against 100 mM NaCl for 2 days, against 25% ethanol for 1 day, and then against distilled water for 1 day, followed by freeze-drying.

For HA-P-PEI synthesis, HA-AEM was synthesized using the same method used for HA-PEI, whereas PEI–oligopeptide was synthesized, as follows: PEI and GPLGLAGC were dissolved in distilled water, followed by the addition of EDC and N-hydroxysuccinimide to the peptide solution and mixing with the PEI solution (pH 6.0) with gentle stirring at 25 °C for 4 h. The product solution was dialyzed (35 kDa) against distilled water, lyophilized, and stored at −20 °C. HA-AEM (20 mg) and PEI-PLG (400 mg) were then dissolved in 0.2 M phosphate-buffered saline (PBS; pH 7.4), stirred for 4 h at 25 °C, dialyzed for 48 h, and then lyophilized. Fourier-transform infrared spectroscopy and ^1^H nuclear magnetic resonance (NMR) experiments were conducted to confirm the composition of the materials.

### Micelle preparations and characterization

siRNA micellar complexes were prepared by gently blending 10 μL of the siRNA solution (20 μM in diethylpyrocarbonate water) with 90 μL of the polymer solution (0.1 μg/μL in diethylpyrocarbonate water), followed by incubation at 25 °C for 30 min. Samples of polymer/siRNA complexes were loaded and electrophoresed on 1.0% agarose gels containing ethidium bromide (1 μg/mL) at 120 V for 15 min in a Tris-borate-EDTA buffer solution. Particle size and surface zeta potential of the siRNA-condensed micellar complexes were measured using dynamic light scattering (DLS; Nano-ZS90; Malvern Instruments, Malvern, UK) at 25 °C after dilution of the micelles with distilled water. Transmission electron microscopy (TEM; JEM-2100 Plus; JEOL, Tokyo, Japan) was used to observe the size and morphology of the nanoparticles, in which the samples were negatively stained with sodium phosphotungstate. To evaluate the MMP-2 sensitivity of the HA-P-PEI/siRNA nanoparticles, 0.1 mL of the nanoparticles (0.1 μg/mL) and 0.1 mL of the MMP-2 solution in HEPES buffer (0.6 μg/mL, pH 7.4) were incubated at 37 °C. DLS was performed at 0 h, 2 h, 6 h, 16 h, 24 h, and 48 h after incubation, and TEM was performed after 6 h of incubation.

### Serum stability of the siRNA-loaded nanoparticles

The serum stability of the complexes was determined by incubating with serum solution. Briefly, the PEI/siRNA, HA-PEI/siRNA, and HA-P-PEI/siRNA complexes were prepared at N/P = 24:1 (N/P ratio: the ratios of moles of the amine groups of cationic polymers to those of the phosphate ones of RNA), and then incubated with fetal bovine serum (FBS; 1:1 v/v) at 37 °C. A total of 1 μL of heparin solution (12 kDa,12500 IU; Tianjin Biochem Pharmaceutical Co., Ltd., Tianjin, China) was added to de-complex the siRNA from the polymer after 0 h, 1 h, 3 h, 6 h, 8 h, and 24 h of incubation, at which time the samples were visualized by gel electrophoresis, as described.

### Cell culture and detection of CD44, PD-L1, and MMP-2 levels in NCI-H1975 cells

The H1975 cell line was obtained from the Cell Bank of Chinese Academy of Sciences (https://www.cellbank.org.cn/xibaoximulu.php) and grown in Roswell Park Memorial Institute (RPMI) 1640 media (Hyclone, Logan, UT, USA) with 10% FBS at 37 °C in a 5% CO_2_ atmosphere. The cells were then digested with trypsin (0.25%), collected, washed with cold PBS, and stained with the CD44-APC and PD-L1-PE monoclonal antibodies for 20 min at room temperature. PBS (500 μL) was then added and detected using a flow cytometer (Invitrogen, Carlsbad, CA, USA). Western blot was used to determine MMP-2 levels in H1975 cells.

### Cell viability assay

To test polymer cytotoxicity against the cells, a 3-(4,5-dimethylthiazol-2-yl)-2,5-diphenyltetrazolium bromide (MTT) assay was conducted. Briefly, H1975 cells under favorable growth conditions were seeded into 96-well plates (4000 cells/well). After 24 h of adherent growth, cells were incubated with different concentrations of PEI, HA-PEI, and HA-P-PEI solutions for 48 h, after which 20 μL of MTT (5 mg/mL) was added to each well and incubated for 3 h. MTT formazan precipitate was dissolved in 100 μL DMSO, and the absorbance was measured at 570 nm using a microtiter plate luminometer (ReadMax 1200; Flash, Shanghai, China).

### Cellular uptake of complexes in vitro

Flow cytometry (Invitrogen) and fluorescence microscopy (Eclipse Ts2R; Nikon, Tokyo, Japan) analyses were used to evaluate cellular uptake of the polymer/siRNA complexes. For flow cytometry, FAM-labelled siRNA was loaded into the micelles, and 12 × 10^4^ H1975 cells/well were inoculated into 12-well plates and incubated in RPMI 1640 medium containing 10% FBS until 70–80% confluence. The medium was then replaced with fresh medium, and free FAM-siRNA, PEI/FAM-siRNA, HA-PEI/FAM-siRNA, and HA-P-PEI/FAM-siRNA (N/P = 24:1; 100 nM) complexes were added and cultured at 37 °C for 4 h. Untreated cells were used as negative controls. The cells were then collected after trypsinization and washed three times with cold PBS, followed by measurement of fluorescence intensity. The cells were then resuspended in 500 μL PBS and analyzed using flow cytometry (Invitrogen).

Cellular uptake of the complexes was confirmed by microscopy analyses using Cy3-labeled siRNA and the same transfection procedure. After incubating with free Cy3-siRNA, PEI/Cy3-siRNA, HA-PEI/Cy3-siRNA, and HA-P-PEI/Cy3-siRNA for 4 h, the cells were washed three times with cold PBS and stained with Hoechst 33342 (Abcam, Cambridge, UK) for 20 min and then washed with PBS. Photographs were obtained using a fluorescence microscope (Eclipse Ts2R; Nikon).

### Tumor-spheroid penetration

Tumor spheroids can not only simulate the *in vivo* environment but also constitute an intuitive and controllable cell culture. H1975 spheroids were produced using the hanging-drop method. Briefly, 1 × 10^5^ cells from a single-cell suspension were dispersed in 2 mL of RPMI 1640 medium and 1 mL of the 1.2% methylcellulose mixed solvent, followed by dropping the suspensions onto the cover of a dish. After incubating for 72 h, the spheroids grew to 200 μm and were transferred to flat-bottomed 48-well plates pretreated with 2% agarose. To evaluate the penetration efficacy of the nanoparticles, free Cy3-siRNA, PEI/Cy3-siRNA, HA-PEI/Cy3-siRNA, HA-P-PEI/Cy3-siRNA, and HA-P-PEI/Cy3-siRNA (MMP-2-pretreated) were added, and after a 4-h culture, the solution containing the tumor spheroids was collected and centrifuged (300 rpm). The precipitates were washed three times with a cold PBS and transferred into confocal dishes (Wuxi NEST Biotechnology Co., Ltd., Wuxi, China). Photographs at different penetration depths were obtained using a confocal laser scanning microscope (CLSM880; Carl Zeiss, Oberkochen, Germany), and fluorescence intensity was analyzed using ImageJ software (Schneider et al., 2012).

### Gene silencing by the PD-L1 − siRNA complexes in vitro

The gene-silencing efficacy of PD-L1–siRNA in NCI-H1975 cells was evaluated by reverse transcriptase-polymerase chain reaction (RT-PCR). H1975 cells (24 × 10^4^ cells/well) were inoculated into 6-well plates and incubated at 37 °C for 24 h. The medium was then replaced with a fresh complete medium with 10% FBS (1.8 mL), and both HA-PEI/PD-L1–siRNA and HA-PLG-PEI/PD-L1–siRNA complexes (N/P = 24:1; 100 nM) were added. PBS and NC siRNA were used as controls, and Lipofectamine 3000 was used as a positive control according to manufacturer instructions (Invitrogen). After a 6-h incubation, the medium was replaced with a complete medium. After transfection for 24 h, total mRNA was isolated and reverse transcribed using the Evo M-MLV RT kit (Accurate Biotechnology Co., Ltd., Beijing, China) according to manufacturer instructions. RT-PCR was conducted using a PCR system (Q2000A; Hangzhou LongGene Scientific Instruments Co., Ltd., Hangzhou, China) using SYBR qPCR master mix (Vazyme, Nanjing, China). Primers for *glyceraldehyde 3-phosphate dehydrogenase* (*GAPDH*) and (*PD-L1*) were as follows: *GAPDH*-forward, GGAGCGAGATCCCTCCAAAAT and *GAPDH*-reverse, GGCTGTTGTCATACTTCTCATGG; and *PD-L1*-forward, GCCGAAGTCATCTGGACAAGC and *PD-L1*-reverse, GTGTTGATTCTCAGTGTGCTGGTCA. Amplifications were performed over the course of 40 cycles at 95 °C for 10 s and 60 °C for 30 s, with one cycle at 95 °C for 300 s. *GAPDH* used as an internal reference, and data were normalized prior to statistical analysis.

To measure transfection efficacy at the protein level, western blot analysis was conducted after siRNA treatment for 48 h, as described. Cells were lysed with radioimmunoprecipitation assay lysis buffer (Beyotime, Beijing, China), and 10% sodium dodecyl sulfate-polyacrylamide gel electrophoresis was used to separate the proteins (Bio-Rad Laboratories, Richmond, CA, USA). The proteins were transferred onto nitrocellulose membrane and incubated in 5% skim milk for 1 h, followed by incubation of the membranes with anti-PD-L1 (1:1000; Abcam) and anti-calnexin (1:1000; Abcam) overnight at 4 °C. The membranes were then washed three times with PBS containing Tween-20 and incubated with the secondary antibody (anti-rabbit immunoglobulin G; 1:5000; Abways) for 1 h.

### Statistical analysis

Statistical analysis was performed using Prism GraphPad software (v.6.0; GraphPad Software, La Jolla, CA, USA) software and the Student’s *t*-test. *p* < 0.05 was considered significant.

## Results and discussion

### Synthesis and characterization of HA-PEI and HA-PLG-PEI

**Figure 1.**
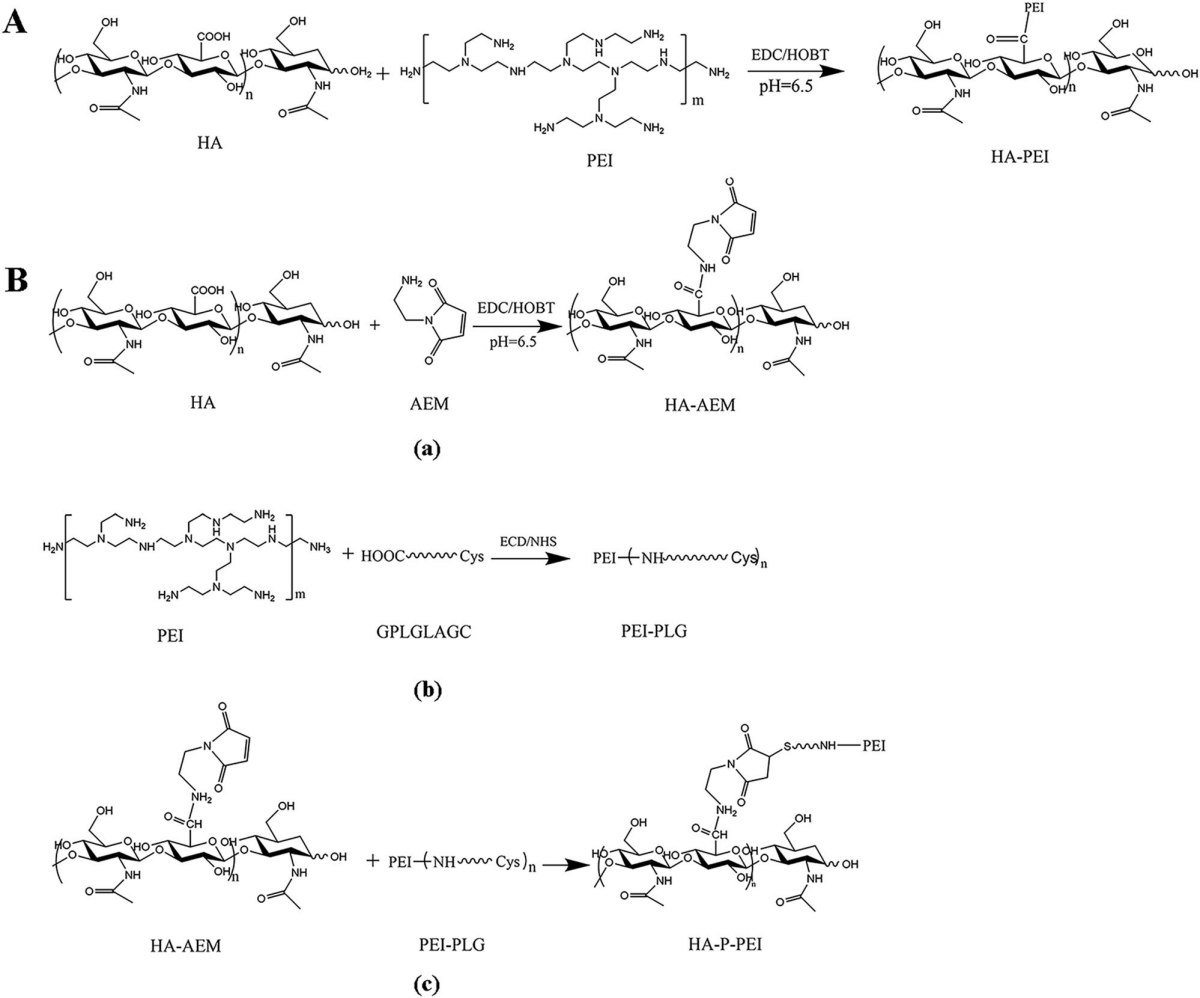
Synthesis of the HA-PEI (A) and HA-P-PEI (B) complexes.

**Figure 2.**
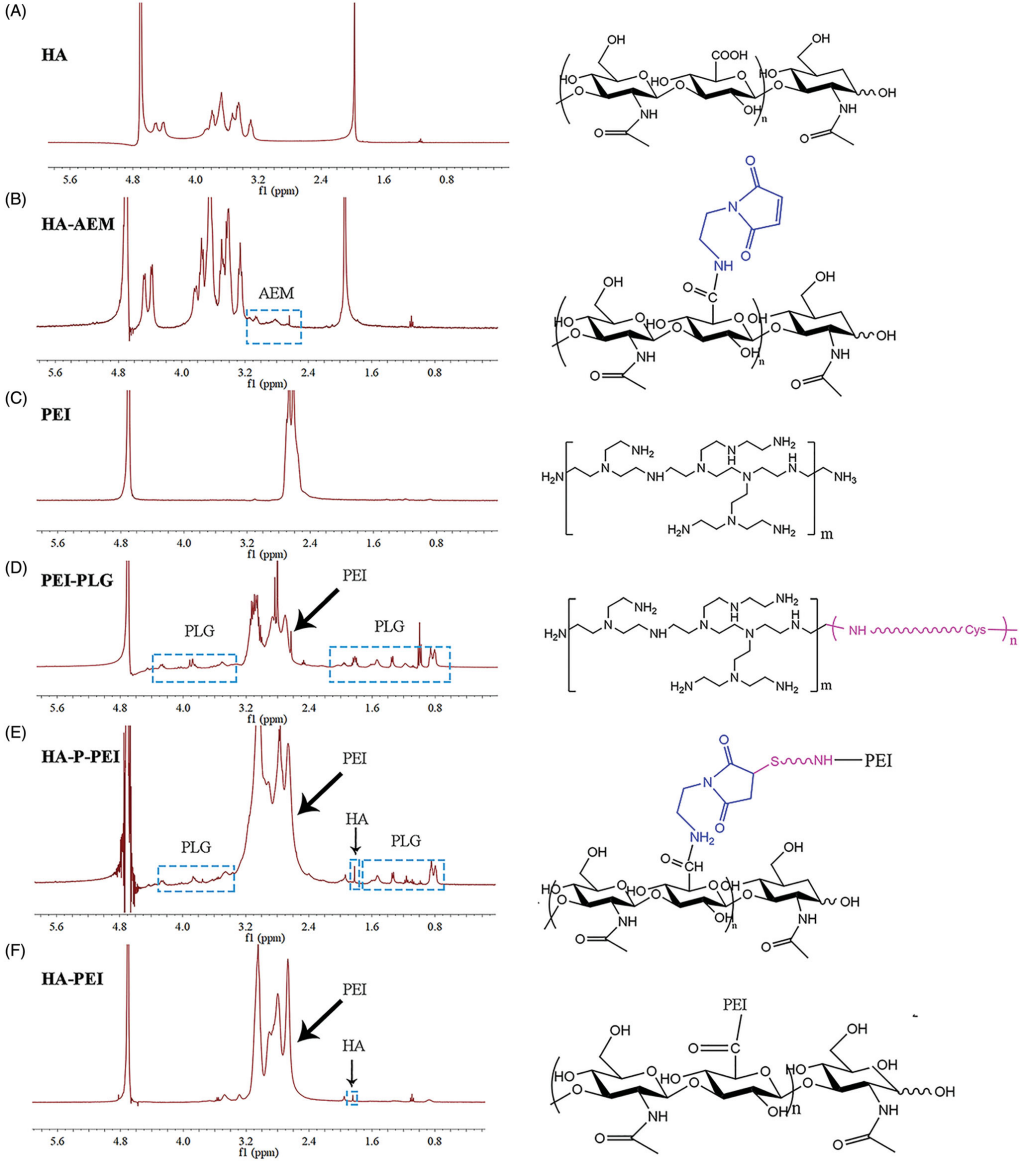
^1^H NMR of HA (A), HA-AEM (B), PEI (C), PEI-PLG (D), HA-P-PEI(E), and HA-PEI (F) in D_2_O.

As shown in Figure 1(A), the HA-PEI conjugate was synthesized by attaching the amino group of PEI (25 kDa) to the carboxyl group of HA (20 kDa) using EDC/HOBt as the catalyst. ^1^H NMR results (Figure 2(F)) demonstrated the successful synthesis of HA-PEI according to the methyl peak of HA at 1.9 and the representative peaks of PEI from 2.5 to 3.2. The calculated weight ratio of HA to PEI was 1:20, and the HA-P-PEI conjugate comprised a 25-kDa PEI backbone, a Gly-PLGLAG-Cys linker, and HA-AEM. As shown in Figure 1(B), HA-P-PEI was synthesized using a three-step reaction. First, HA-AEM was synthesized by attaching the amino group of AEM to the carboxyl group of HA using EDC/HOBt as the catalyst. Signal peaks between 2.6 and 3.2 of AEM are shown in Figure 2(B). PEI-PLG was then prepared by linking the carboxyl group of the peptide to the amino group of PEI, with the signal peaks of PLG (*δ* 0.6–2.2 and 3.2–4.4) are shown in Figure 2(D). Finally, HA-P-PEI was obtained by an addition reaction through the thiol group on PEI-PLG and the double bond on HA-AEM. ^1^H NMR results (Figure 2(E)) showed the characteristic signals of HA (*δ* 1.9), PLG (*δ* 0.6–2.2, 3.2–4.4), and PEI (*δ* 2.4–3.2), indicating successful synthesis of HA-PLG-PEI. The calculated weight ratio of HA to PEI was 1:11. Moreover, the infrared spectrum (Figure S1) showed representative absorption peaks of the hydroxyl groups of HA at 3380 nm for both HA-PEI and HA-P-PEI. Additionally, we observed stretching vibration peaks of NH in PEI between 3100 and 3300 nm, with the signal becoming weaker following conjugation of HA with PEI.

### Characterization of the HA-P-PEI/siRNA complexes

**Figure 3.**
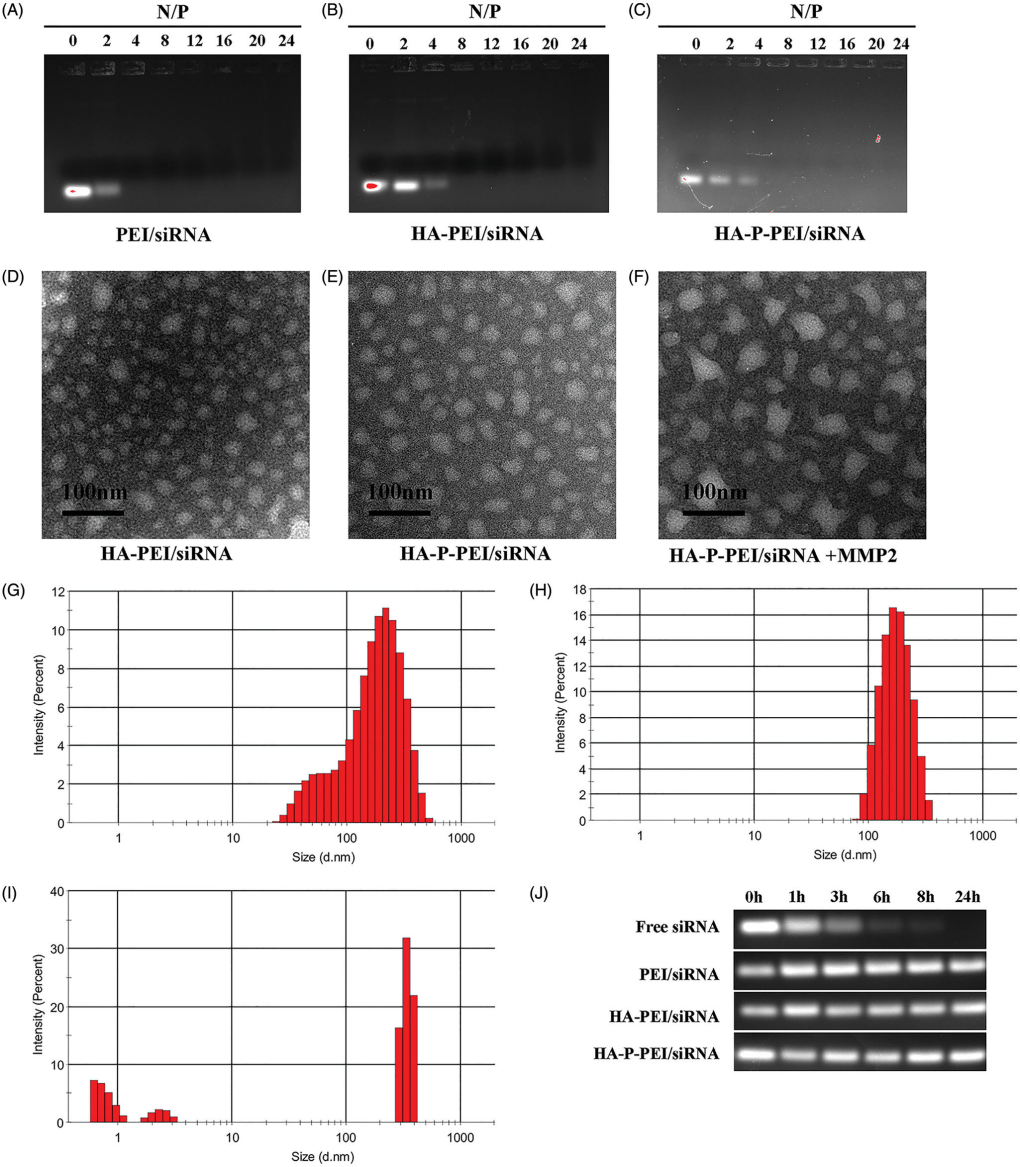
Characterization of HA-P-PEI/siRNA nanoparticles. Gel-retardation assay of PEI/siRNA (A), HA-PEI/siRNA (B) and HA-P-PEI/siRNA (C) at different N/P ratios. TEM and DLS results for the HA-PEI/siRNA (D, G) and HA-P-PEI/siRNA nanoparticles before (E, H) and after (F, I) MMP-2 treatment for 6 h (J) Serum stability of Free siRNA, PEI/siRNA, HA-PEI/siRNA and HA-P-PEI/siRNA.

The ability of the polymers to encapsulate the siRNA was assessed by agarose gel electrophoresis (Figure 3(A–C)), where siRNA migration was completely retarded at N:P ratios >4 in the PEI/siRNA complexes (the ratios were ∼8 for the HA-PEI/siRNA and HA-P-PEI/siRNA complexes). These results suggested that the nanocomplex successfully encapsulated siRNA and formed a stable HA-P-PEI/siRNA nanocomplex, with no siRNA bands observed by gel electrophoresis.

We then evaluated the dispersion and morphology of the HA-PEI/siRNA and HA-P-PEI/siRNA complexes by TEM (Figure 3(D,E)). The results suggested that most of the polyplexes were nearly spherical, indicating that the HA-PEI and HA-P-PEI polymers could interact with siRNA through electrostatic attraction and condense the siRNA to form nanocomplexes. The size and zeta potential of the complexes were measured by DLS (Figure 3(G,H); Table 1), revealing that the sizes of the HA-PEI/siRNA and HA-P-PEI/siRNA nanoparticles (N/P = 24:1) were ∼200 nm. After exposure of HA-P-PEI/siRNA to MMP-2 for 6 h, TEM analysis (Figure 3(F)) showed that the original shape of the particles was fractured, resulting in large and small particles. Additionally, DLS results (Figure 3(I)) showed that some of the nanoparticles turned into very small particles (<10 nm), with the number of small particles (<100 nm) significantly increasing from 7.1 to 25.6% (*p* < .05) following exposure to MMP-2 (Figure S2). This phenomenon is consistent with a previous report demonstrating that MMP-2 treatment resulted in ∼20% of PEG shells detaching from the surface of particles along with slightly larger sizes (Ke et al., 2017). Furthermore, zeta-potential measurements showed that the surface charge of HA-PEI/siRNA and HA-P-PEI/siRNA decreased from 37.6 to 16.4 mV and 6.89 mV, respectively, following MMP-2 treatment.

**Table 1.** Summary of particle size, polydispersity index (PDI) and zeta potential of nanoparticles.

	Size (nm)	PDI ± SD	Zeta (mV)
PEI/siRNA	919.2 ± 107.2	0.551 ± 0.393	37.6 ± 0.569
HA-PEI/siRNA	209.4 ± 39.69	0.359 ± 0.036	16.4 ± 2.48
HA-P-PEI/siRNA	186.4 ± 12.31	0.351 ± 0.100	6.89 ± 0.736

We then assessed the stability of the complexes by incubating free siRNA, PEI/siRNA, HA-PEI/siRNA, and HA-P-PEI/siRNA with FBS at 37 °C. Gel-retardation studies to observe siRNA degradation at various time intervals (Figure 3(J)) revealed that the polymer/siRNA complexes protected the siRNA for up to 24 h, whereas no siRNA was observed in the free-siRNA samples after 3 h.

### CD44, PD-L1, and MMP2 expressions in NCI-H1975 cells

**Figure 4.**
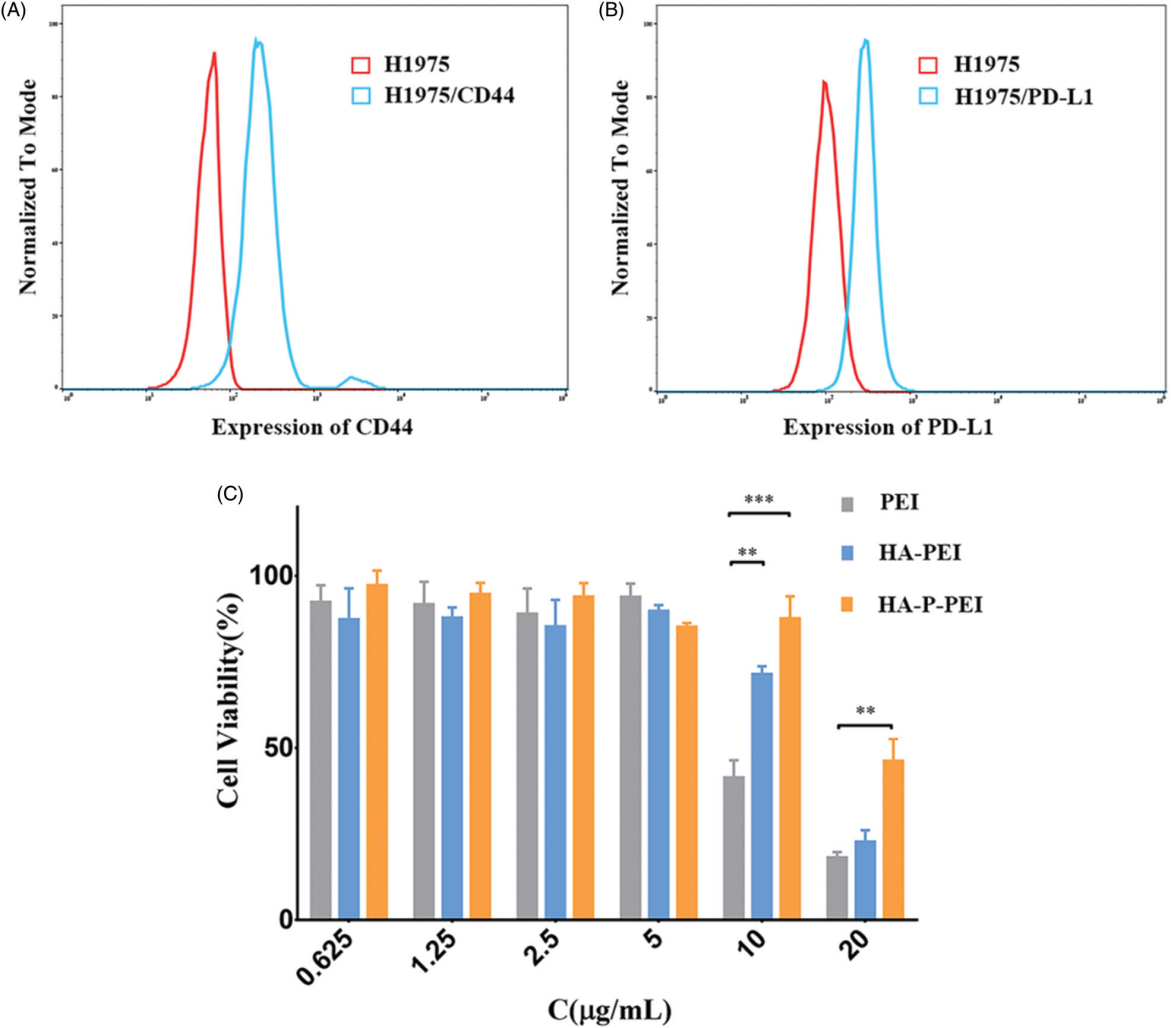
CD44 (A) and PD-L1 (B) expression in H1975 cells according to flow cytometry. MTT assay results (C) following treatment with PEI, HA-PEI, and HA-P-PEI solutions for 48 h.

A previous study reported that HA strongly binds CD44 receptors overexpressed on many cancer cells, which makes them capable of increasing the cellular uptake of the nanoparticles (Dosio et al., 2016). Additionally, studies revealed that MMPs are overexpressed in various types of tumors, where their sensitive substrates can be used to develop tumor-microenvironment-responsive nanoparticles to achieve better tumor diagnosis or treatment (Olson et al., 2010; Mansour et al., 2003). Because the present study aimed to develop an HA-conjugated, MMP-2 sensitive PD-L1–siRNA-delivery system, detection of CD44, MMP-2, and PD-L1 expression was required. We found that both CD44 and PD-L1 were overexpressed in NCI-H1975 cells (Figure 4(A,B)), whereas western blot confirmed that MMP-2 was also overexpressed in these cells (Figure S3).

### Cytotoxicity of the HA-PEI and HA-P-PEI polymers

To determine whether HA–PEI coupling can reduce PEI cytotoxicity, we performed MTT assays on NCI-H1975 cells. After a 48-h incubation, the viability of NCI-H1975 cells was significantly lower in the presence of PEI alone relative to that with HA-PEI and HA-P-PEI (10 μg/mL each) (Figure 4(C)). Moreover, the rate of proliferative inhibition by PEI was 58.62%, whereas that by HA-PEI was 28.57% (*p* < 0.01 vs. PEI), and that by HA-P-PEI was 12.37% (*p* < 0.001 vs. PEI). At a higher polymer concentration (20 μg/mL), the inhibition rate by PEI alone reached 81.86%, whereas that for HA-PEI was 77.24% and that for HA-P-PEI was 53.76% (*p* < 0.01 vs. PEI). These results indicated that coupling of HA-AEM with PEI-PLG significantly reduced PEI cytotoxicity. A previous study reported that the positively charged PEI induced cell death and caused cytotoxicity both *in vitro* and *in vivo*, thereby greatly hindering its clinical application (Shao et al., 2015); however, the mechanism of PEI toxicity remains poorly understood. Several PEI-interacting proteins, including heat-shock proteins, glutathione-S-transferases, and protein disulfide isomerases (involved in apoptosis), have been identified in PEI-specific toxicity pathways (Khansarizadeh et al., 2016). Coupling PEI with negatively charged HA promotes electrostatic neutralization of PEI, and as the HA: PEI molar ratio increased, zeta-potential values decreased due to the addition of HA (Kim et al., 2011). Moreover, we found that the weight ratio of PEI in HA-P-PEI (91.67%) was lower than that in HA-PEI (95.24%), resulting in a lower zeta potential for HA-P-PEI/siRNA nanoparticles relative to that for HA-PEI/siRNA at the same N/P and resulting in the decreased toxicity of HA-P-PEI relative to HA-PEI.

### Cellular uptake of the nanocomplexes

**Figure 5.**
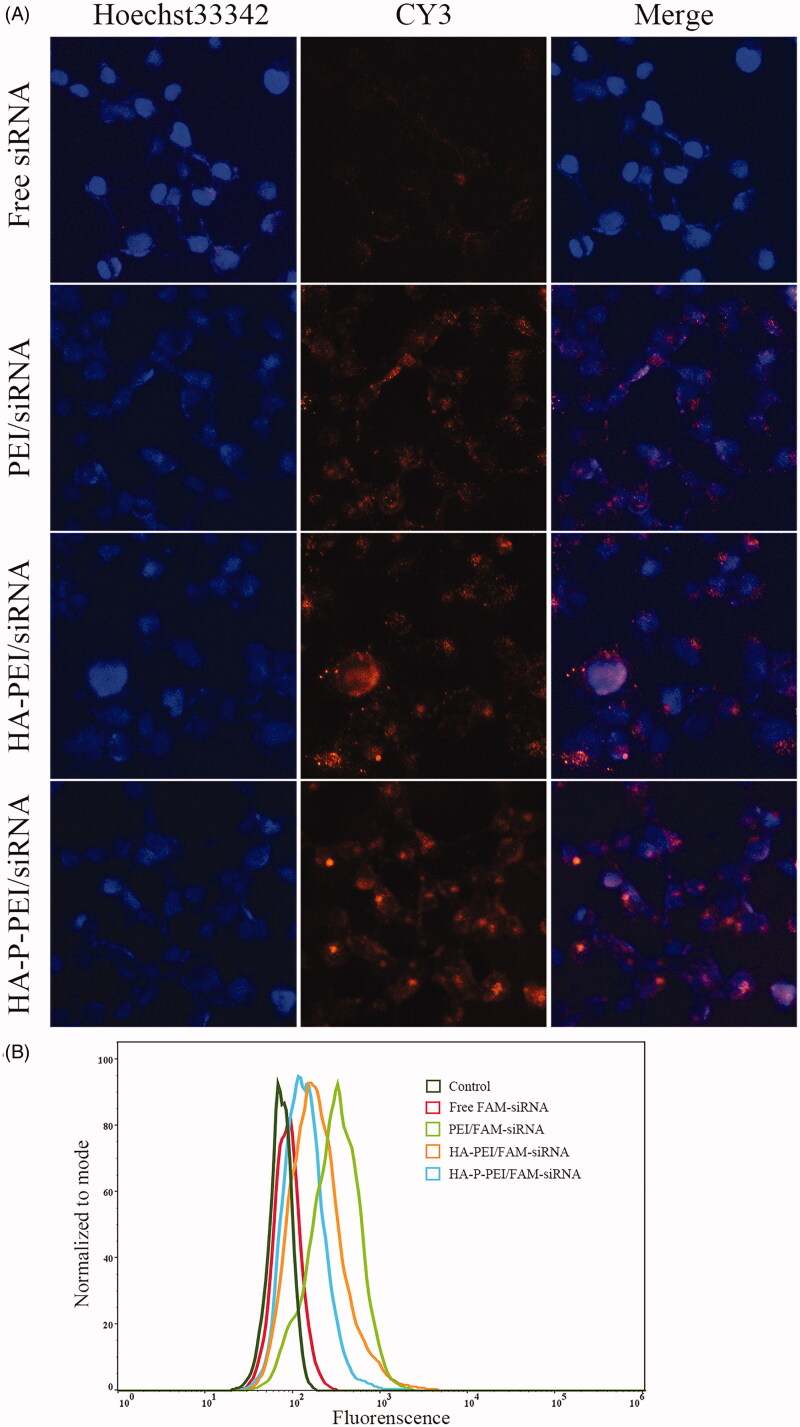
*In vitro* intracellular distribution and cell-uptake efficiency. (A) Intracellular distribution of HA-P-PEI/CY3-siRNA nanoparticles in H1975 cells at 4 h. Red, fluorescence-siRNA; blue, Hoechst 33342. (B) The uptake efficiency of HA-P-PEI/FAM-siRNA nanoparticles in H1975 cells at 4 h.

We then investigated cellular uptake of the nanocomplexes by fluorescence microscopy and flow cytometry using Cy3-conjugated siRNA (red fluorescence) loaded into different polymers and incubated in NCI-H1975 cells for 4 h at 37 °C. Figure 5(A) shows that significant fluorescent signals were observed in polymer/Cy3-siRNA-treated cells, whereas red fluorescent signals were mostly distributed in cell nuclei. By contrast, few free Cy3-conjugated siRNAs penetrated the cells. To quantitatively evaluate the delivery efficacy into the cells, flow cytometric studies were conducted, revealing the highest cellular uptake of FAM-siRNA in the PEI/FAM-siRNA group. Similarly, we observed an increase in FAM-siRNA uptake in the HA-P-PEI/FAM-siRNA group relative to the free FAM-siRNA group (Figure 5(B)). Moreover, the fluorescence intensity in HA-PEI/FAM-siRNA-treated cells was weaker than that in the PEI/FAM-siRNA-treated group and stronger than that in the HA-P-PEI/FAM-siRNA-treated group. This might be attributed to the decreased zeta potential as a result of PEI coupling with HA, as HA reportedly has a strong negative impact on transfection efficiency (van de Wetering et al., 1999).

### Penetration of the nanocomplexes into H1975 tumor spheroids

**Figure 6.**
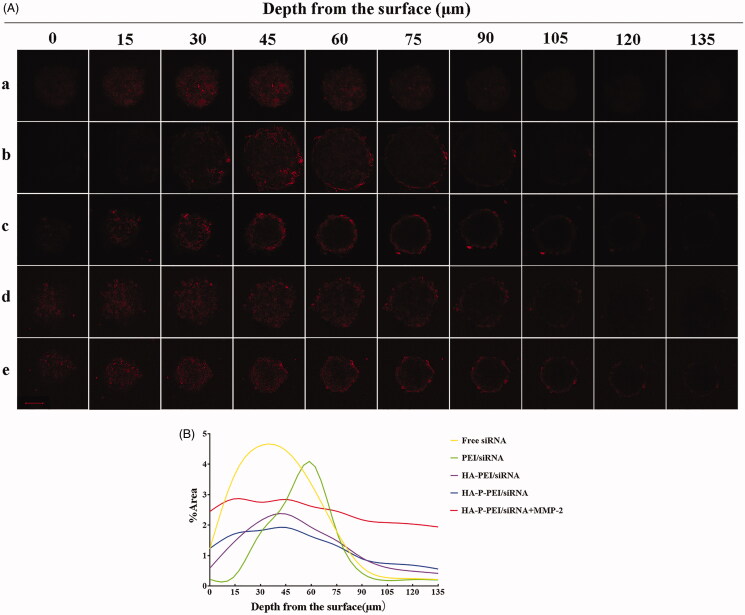
Penetration of nanoparticles into H1975 tumor spheroids. (A) Fluorescence distribution across different depths after being treated with free CY3-siRNA (a), PEI/CY3-siRNA (b), HA-PEI)/CY3-siRNA (c), HA-P-PEI/CY3-siRNA (d), and HA-P-PEI/CY3-siRNA (MMP-2 pretreatment) (e). Magnification: 200×. (B) Semi-quantitative intensity across different sections of H1975 tumor spheroids.

The tumor-spheroid model can precisely reflect the penetration of nanoparticles into deeper regions of solid tumors, making it a more realistic *in vivo* simulation. The effects of different formulations at different depths in H1975 tumor spheroids are shown in Figure 6(A). Free siRNA and PEI/siRNA showed increased penetration depths of 30 µm and 60 μm, which might be attributed to the small size of the free siRNA and the positive charge of PEI. Additionally, the fluorescence intensity of HA-PEI/siRNA and HA-P-PEI/siRNA also decreased inside the spheroid, which might have been due to the coupling of negatively charged HA and the relatively large particle size. Moreover, HA-P-PEI/siRNA nanoparticles pretreated with MMP-2 showed increased fluorescence intensity at deeper levels in the spheroids. Further analysis of the effect of MMP-2 sensitivity and the fluorescence of the nanoparticles at different depths showed that after the addition of MMP-2, penetration of the HA-P-PEI/siRNA nanoparticles into the spheroids significantly increased relative to that of the HA-P-PEI/siRNA nanoparticles without MMP-2 treatment (Figure 6(B)). Therefore, these results support the hypothesis that shrinkage of the large particles to smaller particles can facilitate the penetration process.

Hyaluronidases that degrade HA have been implicated in tumor progression and metastasis, as well as angiogenesis, with previous studies revealing that hyaluronidases can be used to develop HA-based drug-delivery systems (Liu, Hu, et al., 2019; Luo et al., 2019; Yu et al., 2020). However, in the present study, H1975 tumor penetration showed decreases in the fluorescence intensity of HA-PEI/siRNA inside the spheroid relative to PEI/siRNA, demonstrating that HA detachment via hyaluronidase was insufficient to degrade HA and expose PEI. There are three reasons explaining this phenomenon. First, the molecular weight of HA in this study was only 20 kDa, which is almost the same as degraded human HA fragments. A previous report revealed that hyaluronidase-1 and -2 cleave HA to small (<20 kDa) fragments (Patel et al., 2002). Second, hyaluronidase expression in H1975 cells is too low to degrade HA. Third, the type of hyaluronidase expressed in H1975 cells is not enzymatically active.

We found that penetration of HA-PEI/siRNA nanoparticles into the tumor spheroid was hindered by particle size and the negatively charged HA; therefore, we added an MMP-2-sensitive linker to synthesize HA-P-PEI. The results showed that penetration of the HA-P-PEI/siRNA nanoparticles into the spheroids significantly increased relative to nanoparticles without MMP-2 treatment and HA-PEI/siRNA nanoparticles. This was attributed to the size of the particles (<100 nm), which decreased following exposure to MMP-2. Future studies will be conducted on the expression of hyaluronidase in H1975 cells and the release of HA from HA-PEI/siRNA nanoparticles.

### RT-PCR and Western blot verification of PD-L1 knockdown

**Figure 7.**
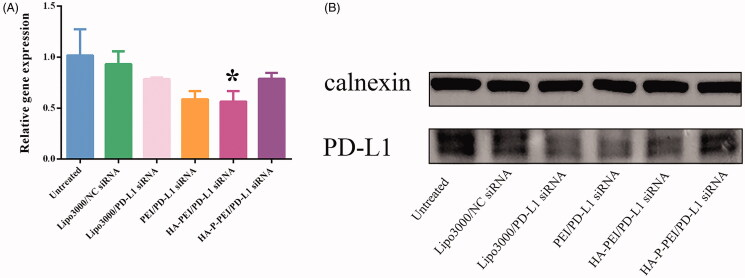
*In vitro* gene-silencing efficiency in H1975 cells. Relative levels of PD-L1 mRNA (A) and protein (B) in H1975 treated with PBS or transfected with Lipo3000/NC siRNA, Lipo3000/PD-L1–siRNA, PEI/PD-L1–siRNA, HA-PEI/PD-L1–siRNA, or HA-P-PEI/siRNA and determined by qRT-PCR and western blot.

We then determined the PD-L1–siRNA effectiveness in NCI-H1975 cells. RT-PCR analysis of NCI-H1975 cells transfected with Lipo3000/PD-L1–siRNA (positive control), PEI/PD-L1–siRNA, HA-PEI/PD-L1–siRNA, HA-P-PEI/PD-L1–siRNA, and Lipo3000/NC siRNA for 24 h revealed decreased levels of *PD-L1* mRNA in cells treated with Lipo3000/PD-L1–siRNA, PEI/PD-L1–siRNA, HA-PEI/PD-L1–siRNA, and HA-P-PEI/PD-L1–siRNA relative to the NC (Figure 7(A)). Specifically, HA-PEI/PD-L1–siRNA transfection decreased *PD-L1* mRNA levels by up to 45% relative to the NC group (*p* < 0.05). Western blot analysis confirmed these findings (Figure 7(B)), with the HA-PEI/PD-L1–siRNA-treated group also showing clear downregulation of PD-L1 to levels similar to those in PEI/PD-L1–siRNA- and the Lipo3000/PD-L1–siRNA-treated cells along with observation of high serum stability of the nanoparticles treated with 50% FBS for 24 h.

Additionally, although HA-PEI/PD-L1–siRNA demonstrated higher efficacy in attenuating *PD-L1* expression relative to HA-P-PEI/PD-L1–siRNA, the tumor penetration of HA-PEI was weaker than that of HA-P-PEI (with the treatment of MMP-2). The addition of MMP-2 shrunk the HA-P-PEI/PD-L1–siRNA nanoparticles, resulting in deeper penetration of tumor spheroids. Furthermore, the HA-P-PEI nanoparticles demonstrated decreased cytotoxicity than the HA-PEI nanoparticles, suggesting their potential therapeutic superiority.

## Conclusions

In this study, we developed and evaluated an MMP-2-sensitive siRNA-delivery system. The results showed that the size of the HA-P-PEI/siRNA nanoparticles decreased from 186.4 nm to <10 nm following the addition of the MMP-2 linker. We demonstrated that HA-P-PEI/siRNA could be effectively taken up by H1975 cells, and western blot showed that HA-P-PEI/PD-L1–siRNA successfully downregulated PD-L1 levels in these cells along with high penetrative ability into tumor spheroids. These findings indicated that the HA-P-PEI nanoparticles were superior in their ability to support siRNA penetration into solid tumors, although additional studies are necessary to further enhance transfection efficiency and reduce toxicity.

## Supplementary Material

Supplemental Material
