# Supplementary material for: A size-shrinkable matrix metallopeptidase-2-sensitive delivery nanosystem improves the penetration of human programmed death-ligand 1 siRNA into lung-tumor spheroids
Source: Drug Deliv. 2021 Jun 2;28(1):1055–66. doi: 10.1080/10717544.2021.1931560 (PMC8183518; doi:10.1080/10717544.2021.1931560)
Supplement: Supplemental Material [file IDRD_A_1931560_SM4597.docx]

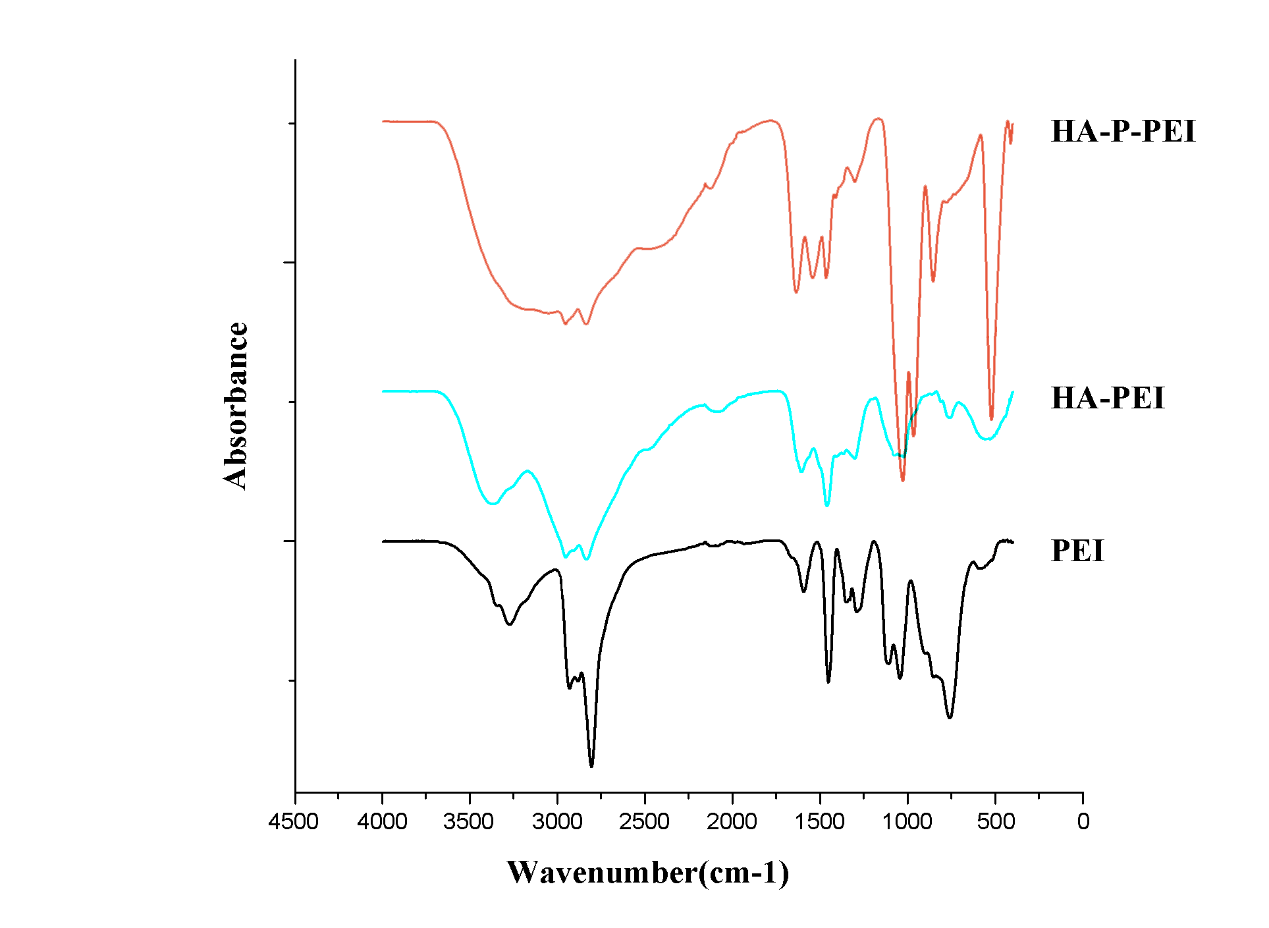


Figure S1. FT-IR spectrum of PEI, HA-PEI and HA-P-PEI.


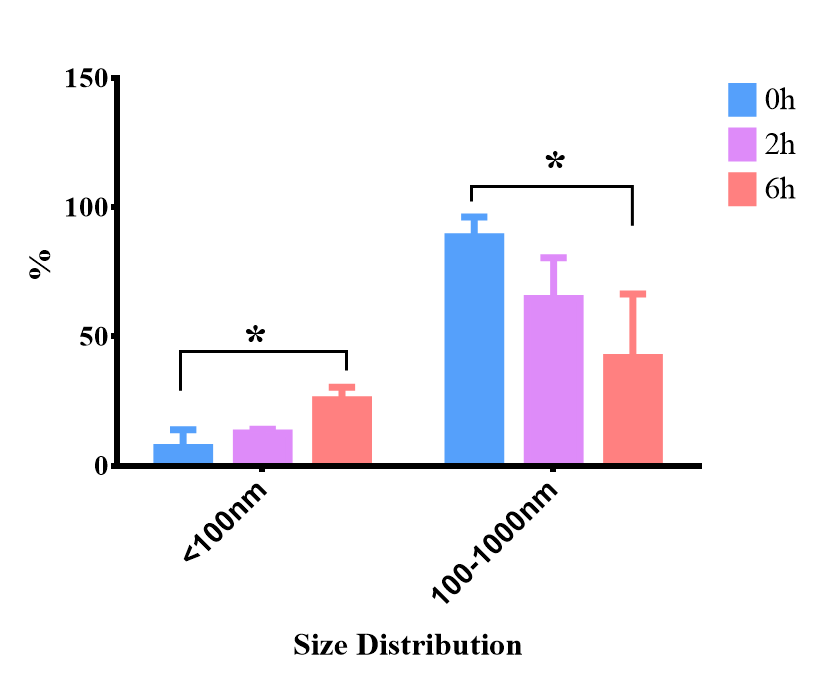


Figure S2. Size change of HA-P-PEI/siRNA particles after exposing in MMP-2


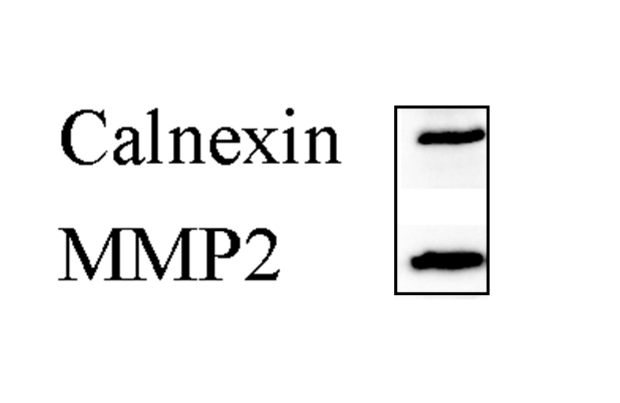


Figure S3. MMP-2 expressing in the NCI-H1975 cells.
